# Cost-effectiveness of first-line aumolertinib versus osimertinib for advanced epidermal growth factor receptor-mutated non-small cell lung cancer in China

**DOI:** 10.3389/fpubh.2026.1858476

**Published:** 2026-09-22

**Authors:** Yuhua Wu, Qingqing Hu, Fengqing Wang, Lejun Zhang, Miaolian Wu, Zhengqiang Hu

**Affiliations:** 1 Department of Pharmacy, the Fourth Affiliated Hospital of School of Medicine, and International School of Medicine, International Institutes of Medicine, Zhejiang University, Yiwu, China; 2 Department of Clinical Research Center, the Fourth Affiliated Hospital of School of Medicine, and International School of Medicine, International Institutes of Medicine, Zhejiang University, Yiwu, China; 3 Research Center for Clinical Pharmacy, Zhejiang University, Hangzhou, Zhejiang, China; 4 Department of Pharmacy, The Children's Hospital, Zhejiang University School of Medicine, National Clinical Research Center for Child Health, Hangzhou, Zhejiang, China

**Keywords:** aumolertinib, cost-utility analysis, EGFR tyrosine kinase inhibitor, EGFR-mutated non-small cell lung cancer, osimertinib

## Abstract

**Objective:**

To evaluate the cost-effectiveness of aumolertinib versus osimertinib as first-line treatment for advanced epidermal growth factor receptor (EGFR)-mutated non-small cell lung cancer (NSCLC) from the perspective of the Chinese healthcare system, and to explore economic outcomes across clinically relevant subgroups.

**Methods:**

A partitioned survival model with three health states was developed over a 10-year horizon with a 28-day cycle length. Clinical inputs were derived from indirect evidence based on the AENEAS and FLAURA trials. In the base-case analysis, the progression-free survival (PFS) effect was informed by a published simulated treatment comparison, whereas the overall survival (OS) hazard ratio for aumolertinib versus osimertinib was conservatively set at 1.00. Costs and health outcomes were discounted at 5% annually. Outcomes included total costs, quality-adjusted life-years (QALYs), and incremental net monetary benefit (iNMB) at a willingness-to-pay threshold of 298,995 Chinese Yuan (CNY) per QALY. One-way sensitivity, probabilistic sensitivity, scenario, threshold, and exploratory subgroup analyses were performed.

**Results:**

In the overall population, osimertinib was associated with a total cost of 193,447.40 CNY and 1.981 QALYs, whereas aumolertinib was associated with a total cost of 207,484.66 CNY and 2.000 QALYs. Compared with osimertinib, aumolertinib yielded an additional 0.019 QALYs at an additional cost of 14,037.26 CNY, resulting in an iNMB of −8,307.60 CNY. Thus, aumolertinib was not economically favorable in the base-case analysis. At the prespecified threshold, the probability of being cost-effective was 0.58 for osimertinib and 0.42 for aumolertinib. Scenario and threshold analyses showed that the results were sensitive to drug price and the comparative OS effect. Exploratory subgroup analyses showed differences in the direction of point estimates across subgroups, but uncertainty remained substantial across all subgroups.

**Conclusion:**

Under the current Chinese pricing environment and available indirect comparative evidence, aumolertinib and osimertinib showed broadly similar economic profiles as first-line treatment for advanced EGFR-mutated NSCLC.

## 1 Introduction

Lung cancer remains a major public health burden worldwide, including in China, and non-small cell lung cancer (NSCLC) accounts for the vast majority of cases (1–3). Epidermal growth factor receptor (EGFR) mutations are more common in East Asian populations than in Western populations (4, 5). As a result, advanced EGFR-mutated NSCLC has particular clinical and policy relevance in China. For this molecular subtype, EGFR tyrosine kinase inhibitors (EGFR-TKIs) have substantially improved disease control compared with conventional chemotherapy and have fundamentally changed the first-line treatment landscape (6, 7).

Osimertinib is a third-generation, irreversible EGFR-TKI with activity against both EGFR-sensitizing mutations and the T790M resistance mutation, and it also has clinically meaningful central nervous system (CNS) penetration (8, 9). In the phase III FLAURA trial, first-line osimertinib significantly prolonged progression-free survival and overall survival compared with gefitinib or erlotinib, while maintaining a favorable tolerability profile despite longer treatment exposure (9, 10). These findings established osimertinib as an important first-line treatment option for patients with advanced EGFR-mutated NSCLC.

Aumolertinib is another third-generation EGFR-TKI developed in China (11, 12). In the phase III AENEAS trial, aumolertinib significantly prolonged progression-free survival compared with gefitinib in previously untreated patients with locally advanced or metastatic NSCLC harboring EGFR exon 19 deletion (Ex19del) or L858R mutations, while showing an overall favorable safety profile (12). These results support aumolertinib as another important first-line option in this setting.

However, the increasing availability of effective EGFR-TKIs has also made treatment selection more complex from an economic perspective. In China, long-term targeted therapy may lead to substantial drug expenditures, and the relative value of competing first-line options may be strongly influenced by local pricing and reimbursement conditions. The relative economic burden of aumolertinib and osimertinib remains unclear because no head-to-head randomized trial has compared these two agents. More importantly, most pharmacoeconomic studies conducted in China have evaluated each drug separately against first-generation EGFR-TKIs or other treatment strategies, rather than directly comparing these two third-generation agents (13–17). In addition, earlier economic findings may no longer fully reflect current decision-making because drug prices have changed over time. Therefore, existing evidence is informative but still does not adequately answer a more practical question in contemporary clinical and policy settings: when both aumolertinib and osimertinib are available as first-line options, which treatment provides better economic value within the current Chinese healthcare system?

Accordingly, this study evaluated the cost-utility of aumolertinib versus osimertinib as first-line treatment for advanced EGFR-mutated NSCLC from the perspective of the Chinese healthcare system. In the absence of head-to-head trial data, we integrated currently available indirect evidence from AENEAS and FLAURA. In addition to the overall population, we also explored clinically relevant subgroups to provide evidence that may be more directly relevant for treatment choice and healthcare resource allocation.

## 2 Methods

### 2.1 Patients and treatments

The target population of this study was aligned with the population enrolled in the FLAURA trial (9), namely patients with previously untreated advanced NSCLC harboring EGFR-sensitizing mutations (Ex19del or L858R).

In the model, patients were assumed to receive first-line aumolertinib 110 mg once daily or osimertinib 80 mg once daily, in accordance with the trial regimens and approved labeling (9, 12), until disease progression. After progression, patients could receive platinum-based chemotherapy or another EGFR-TKI as subsequent treatment. The proportion of patients receiving active subsequent treatment and the treatment mix were based on the FLAURA trial. Among patients receiving subsequent therapy, 67.6% received chemotherapy and 28.6% received EGFR-TKIs, whereas the remainder received VEGF inhibitors or other regimens. To simplify the model and facilitate cost estimation, subsequent treatment was restricted to two categories, represented by pemetrexed plus cisplatin and gefitinib, and their proportions were normalized to 0.7 and 0.3, respectively. Patients who did not receive active subsequent treatment, as well as those who progressed again after subsequent treatment, were assumed to receive best supportive care. Assumptions and parameter inputs for post-progression treatment are provided in Supplementary Table 1.

### 2.2 Model overview and structure

A cost-utility analysis was conducted from the perspective of the Chinese healthcare system to compare aumolertinib with osimertinib as first-line treatment for advanced or metastatic EGFR-mutated NSCLC. A partitioned survival model with three mutually exclusive health states was developed: progression-free survival (PFS), progressed disease (PD), and death. This modeling framework is widely used in oncology economic evaluations and reimbursement decision-making (18). The cycle length was 28 days, the time horizon was 10 years, and both costs and health outcomes were discounted at an annual rate of 5%. In accordance with Chinese pharmacoeconomic guidelines (19), the willingness-to-pay (WTP) threshold was set at three times the 2025 gross domestic product per capita in China, namely 298,995 Chinese Yuan (CNY) per quality-adjusted life-year (QALY). In addition to the main analysis in the overall population, exploratory subgroup analyses were conducted in Asian patients, patients with Ex19del, patients with L858R mutation, and patients with CNS metastases.

### 2.3 Clinical inputs

Because no head-to-head randomized trial has directly compared aumolertinib with osimertinib in the first-line setting, relative treatment effects were derived from indirect evidence based on AENEAS and FLAURA (20). Key design and population characteristics of the two source trials are summarized in Supplementary Table 2. The overall-population PFS HR was based on the published anchored simulated treatment comparison (STC) (HR 0.98, 95% CI 0.68–1.42), which used AENEAS patient-level data and published FLAURA aggregate data with first-generation EGFR-TKIs as the common anchor and adjusted for imbalances in observed prognostic factors and potential effect modifiers, including age, smoking history, Eastern Cooperative Oncology Group (ECOG) performance status, EGFR mutation type, and CNS metastasis status (20, 21). This estimate was closely aligned with the conventional Bucher estimate (HR 1.00, 95% CI 0.72–1.39) and with two subsequently published Bayesian NMAs (22, 23) (Supplementary Table 3).

For OS, an HR of 1.00 was used as a neutral base-case assumption. Available indirect OS estimates were imprecise, while supportive Bucher comparisons incorporating updated long-term AENEAS OS data (24) and the FLAURA overall (10) or FLAURA China (25) results yielded HRs close to 1 (Supplementary Table 3). Comparative OS uncertainty was further examined in the threshold analysis.

For exploratory subgroup analyses, the corresponding subgroup-specific PFS and OS curves from FLAURA were used as anchor curves whenever available. However, for the CNS metastases subgroup, no CNS-specific OS Kaplan–Meier curve or mature subgroup OS data were available. Therefore, the OS component for this subgroup was anchored to the overall-population OS curve, whereas the PFS component remained based on the CNS-specific curve. This approach was intended to preserve subgroup differences in disease progression while maintaining consistency with the overall modeling framework. Because subgroup-specific STC estimates were unavailable, subgroup PFS HRs were derived using the Bucher method (26): ln(HR_aumolertinib vs. osimertinib) = ln(HR_aumolertinib vs. comparator) − ln(HR_osimertinib vs. comparator). Based on published evidence (27), the comparator arms in AENEAS (gefitinib) and FLAURA (gefitinib or erlotinib) were assumed to have equivalent efficacy. Owing to the lack of mature and directly comparable OS evidence, the OS HR was conservatively fixed at 1.00 for all exploratory subgroup analyses, consistent with the main analysis. These subgroup analyses should therefore be interpreted as exploratory assessments of subgroup-specific economic patterns rather than confirmatory subgroup economic comparisons.

Because individual patient-level survival data from FLAURA were unavailable for model reconstruction, PFS and OS data were extracted from published FLAURA Kaplan–Meier curves. Kaplan–Meier data points were digitized using GetData Graph Digitizer, and survival data were reconstructed from published curves using established secondary analysis approaches (28, 29). The extracted data were then fitted in R, and several parametric survival functions, including log-normal, log-logistic, gamma, Weibull, and Gompertz distributions, were evaluated to extrapolate survival beyond the observed trial period. The final distributions were selected on the basis of the Akaike information criterion, Bayesian information criterion, and visual inspection. Detailed clinical inputs are shown in Table 1. The selected parametric distributions and their parameters are reported in Supplementary Table 4, and the reconstructed fitted curves are presented in Supplementary Figures 2–6.

**Table 1:** Relative treatment effects.

Analysis set	Relative effect	Value	Range	Distribution	Source
Overall	PFS HR	0.98	0.68–1.42	Log-normal	(20)
Overall	OS HR	1.00	0.80–1.20	Log-normal	Assumption
Asian	PFS HR	0.85	0.58–1.25	Log-normal	(12, 35)
Asian	OS HR	1.00	0.80–1.20	Log-normal	Assumption
Ex19del	PFS HR	0.91	0.59–1.40	Log-normal	(9, 12)
Ex19del	OS HR	1.00	0.80–1.20	Log-normal	Assumption
L858R	PFS HR	1.18	0.70–1.99	Log-normal	(9, 12)
L858R	OS HR	1.00	0.80–1.20	Log-normal	Assumption
CNS metastases	PFS HR	0.81	0.42–1.54	Log-normal	(9, 12)
CNS metastases	OS HR	1.00	0.80–1.20	Log-normal	Assumption

PFS, progression-free survival; HR, hazard ratio; OS, overall survival; CNS, central nervous system.

### 2.4 Costs and utilities

Only direct medical costs were included in the model. These comprised the cost of EGFR mutation testing at model entry; first-line drug acquisition costs and routine follow-up costs in the PFS state, the weighted average cost of subsequent treatment after progression, best supportive care costs in the PD state, terminal care costs at death, and management costs for grade 3 or higher adverse events with an incidence of at least 2% during follow-up. Drug acquisition costs were obtained from the Zhejiang provincial drug procurement platform. For the two first-line oral EGFR-TKIs, the acquisition cost per 28-day cycle was derived from the package procurement price using a per-tablet approach. Patients were assumed to receive the full labeled dose while on first-line treatment (osimertinib 80 mg once daily or aumolertinib 110 mg once daily), corresponding to 100% relative dose intensity. Dose reductions, temporary treatment interruptions, and treatment discontinuation before disease progression were not separately modeled, and first-line drug acquisition costs were applied until disease progression. Detailed derivations of the first-line drug acquisition costs are provided in Supplementary Table 5. The weighted average cost of subsequent treatment was calculated by multiplying the proportion of patients receiving active post-progression treatment by the expected cost of representative subsequent regimens weighted according to the simplified treatment mix. This cost was applied as a one-time cost at the time of progression. For body-surface-area-based chemotherapy costing, a baseline height of 1.64 m and weight of 65 kg were assumed, corresponding to a body surface area of 1.72 m^2^. Patients who did not receive active subsequent treatment, and those who progressed again after subsequent treatment, were assumed to receive best supportive care.

Health utilities were assigned by health state. The values applied to PFS and PD were 0.804 and 0.321, respectively, based on the China-specific estimates reported by Nafees et al. (30), an international utility-elicitation study using the time trade-off method. The utility for death was set to 0. AE-specific disutilities were not separately modeled. Detailed cost and utility inputs are presented in Table 2. All costs were adjusted to 2025 CNY.

**Table 2:** Cost, utility and adverse-event inputs.

Parameter	Value	Range	Distribution	Source
Cost inputs (CNY)				
Osimertinib, per cycle	4635.12	3708.10–5562.14	Gamma	Supplementary Table 5
Aumolertinib, per cycle	5193.44	4154.75–6232.13	Gamma	Supplementary Table 5
Follow-up during PFS, per cycle	1032.48	825.98–1238.99	Gamma	(16)
EGFR mutation testing	2025.29	1620.23–2430.35	Gamma	(16)
Weighted average subsequent treatment cost	2981.76	2285.41–3578.11	Gamma	Calculated
BSC during PD, per cycle	2961.77	2018.83–3364.71	Gamma	(15)
Terminal care	15667.2	12533.76–18800.64	Gamma	(16)
Costs of AE management (CNY)				
Blood creatine phosphokinase increase	313.21	250.57–375.85	Gamma	(16)
Hypertension	16.74	13.39–20.08	Gamma	(16)
Alanine aminotransferase increase	313.21	250.57–375.85	Gamma	(16)
Leukopenia	3318.71	2654.97–3982.45	Gamma	(16)
Pneumonia	11679.59	9343.67–14015.5	Gamma	(16)
Diarrhea	214.99	171.99–257.98	Gamma	(36)
Anemia	3940.18	3152.15–4728.22	Gamma	(36)
Neutrophil count decreased	3318.71	2654.97–3982.45	Gamma	(16)
Probabilities of AEs: aumolertinib arm				
Blood creatine phosphokinase increase	0.070	0.056–0.084	Beta	(12)
Hypertension	0.042	0.034–0.050	Beta	(12)
Alanine aminotransferase increase	0.028	0.022–0.034	Beta	(12)
Leukopenia	0.023	0.018–0.028	Beta	(12)
Probabilities of AEs: osimertinib arm				
Pneumonia	0.030	0.024–0.036	Beta	(10)
Diarrhea	0.030	0.024–0.036	Beta	(10)
Anemia	0.030	0.024–0.036	Beta	(10)
Neutrophil count decreased	0.020	0.016–0.024	Beta	(10)
Utilities				
PFS	0.804	0.536–0.840	Beta	(30)
PD	0.321	0.050–0.473	Beta	(30)

CNY, Chinese Yuan; PFS, progression-free survival; EGFR, epidermal growth factor receptor; BSC, best supportive care; PD, progressed disease; AE, adverse event. All costs are presented in 2025 CNY.

### 2.5 Base-case outcomes

The primary economic outcome was incremental net monetary benefit (iNMB), calculated as iNMB = WTP × ΔQALY − ΔCost. iNMB was selected as the primary decision metric because the expected incremental QALYs were small across several analysis sets, and the incremental cost-effectiveness ratio (ICER) may become numerically unstable or difficult to interpret when incremental effects approach zero. Secondary outcomes included total costs, total QALYs, incremental costs, incremental QALYs, and net monetary benefit (NMB) for each strategy. A positive iNMB indicated that aumolertinib was economically favorable compared with osimertinib at the prespecified WTP threshold.

### 2.6 Sensitivity, subgroup, scenario, and threshold analyses

To assess model robustness, one-way sensitivity analysis and probabilistic sensitivity analysis were performed. In the one-way sensitivity analysis, key parameters were varied individually across their prespecified ranges or confidence intervals to identify the major drivers of the economic results. In the probabilistic sensitivity analysis, probability distributions were assigned to model inputs and Monte Carlo simulations were used to evaluate overall parameter uncertainty. Gamma distributions were assigned to cost parameters, beta distributions to utilities, event probabilities, and proportions, and log-normal distributions to relative treatment-effect parameters. Results were presented as tornado diagrams, cost-effectiveness acceptability curves, and incremental cost-effectiveness scatter plots.

In addition to the main analysis, exploratory subgroup analyses were conducted in Asian patients, patients with Ex19del, patients with L858R mutation, and patients with CNS metastases, using subgroup-specific anchor curves and PFS HRs while retaining the same conservative OS assumption. The analysis sets were defined as follows: overall analysis, FLAURA overall versus AENEAS overall; Asian analysis, FLAURA Asian versus AENEAS overall; Ex19del analysis, FLAURA Ex19del versus AENEAS Ex19del; L858R analysis, FLAURA L858R versus AENEAS L858R; and CNS analysis, FLAURA CNS versus AENEAS CNS.

Scenario analyses were performed to examine the impact of drug pricing on the economic results. Separate 20% price-reduction scenarios were evaluated for osimertinib and aumolertinib, with all other model inputs held constant. A deterministic threshold analysis was also conducted by varying the OS HR for aumolertinib versus osimertinib from 0.40 to 1.30 while holding all other model inputs constant; the OS HR at which iNMB crossed zero was identified as the approximate cost-effectiveness threshold.

### 2.7 Ethics statement

Ethical review and approval were not required for this study in accordance with local institutional requirements because this analysis was based exclusively on published aggregate data and publicly available information, without access to individual patient data or identifiable personal information. Informed consent was therefore not required.

## 3 Results

### 3.1 Base-case analysis

At the prespecified WTP threshold of 298,995 CNY/QALY, osimertinib was associated with a total cost of 193,447.40 CNY, a total of 1.981 QALYs, and a NMB of 398,857.41 CNY. The corresponding values for aumolertinib were 207,484.66 CNY, 2.000 QALYs, and 390,549.81 CNY. Compared with osimertinib, aumolertinib yielded an additional 0.019 QALYs at an additional cost of 14,037.26 CNY, resulting in an iNMB of −8,307.60 CNY. Therefore, in the base-case analysis, aumolertinib was not economically favorable relative to osimertinib, although the difference between the two strategies was small. Detailed results are presented in Table 3.

**Table 3:** Base-case cost-utility results for aumolertinib versus osimertinib.

Treatment	Cost (CNY)	incrCost (CNY)	QALYs	incrQALYs	NMB (CNY)	iNMB (CNY)
Osimertinib	193447.40	-	1.981	-	398857.41	-
Aumolertinib	207484.66	14037.26	2.000	0.019	390549.81	−8307.60

CNY, Chinese Yuan; QALY, quality-adjusted life-year; NMB, net monetary benefit; iNMB, incremental net monetary benefit.

### 3.2 Sensitivity analyses

In the one-way sensitivity analysis, the tornado diagram showed that the six most influential parameters were, in descending order, the PFS HR for aumolertinib versus osimertinib, the cost of aumolertinib, the OS HR, the cost of osimertinib, the utility of the PFS state, and the utility of the PD state. Overall, the economic results were mainly driven by assumptions regarding relative treatment effect and drug acquisition cost. The tornado diagram is shown in Figure 1.

**Figure 1:**
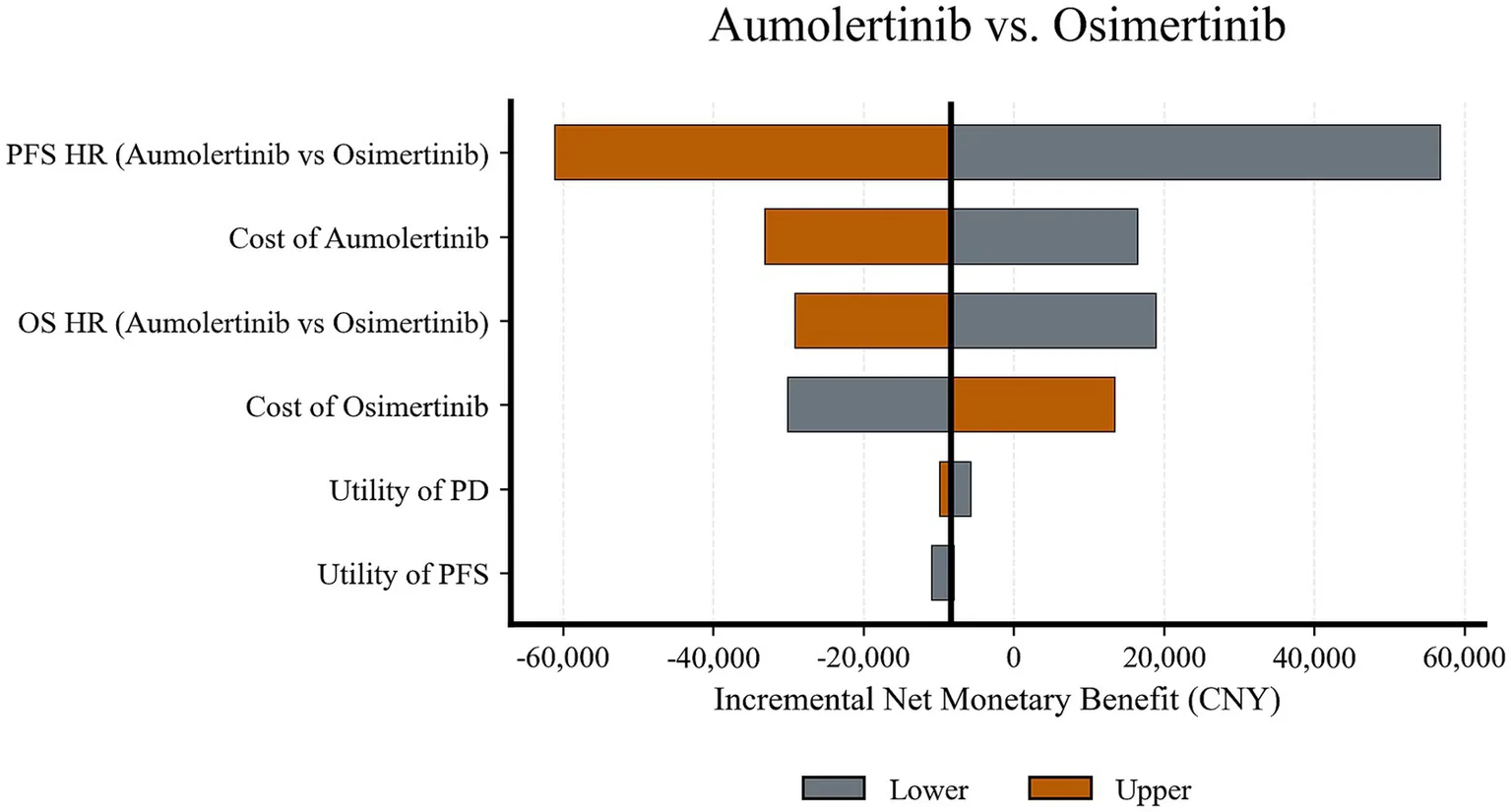
Tornado diagram of one-way sensitivity analysis. Bars show the impact of varying each parameter across its prespecified range on the iNMB of aumolertinib versus osimertinib. The vertical line represents the base-case iNMB. Values to the right of zero favor aumolertinib, whereas values to the left favor osimertinib. PFS, progression-free survival; HR, hazard ratio; OS, overall survival; PD, progressed disease; CNY, Chinese Yuan.

Probabilistic sensitivity analysis indicated that the two strategies had broadly similar economic profiles. Across the evaluated WTP range, the cost-effectiveness acceptability curve generally favored osimertinib, although the gap between the two strategies narrowed as the threshold increased. At the prespecified threshold, the probability of being cost-effective was approximately 58% for osimertinib and 42% for aumolertinib. The incremental cost-effectiveness scatter plot showed that the simulated results were distributed almost evenly on both sides of the cost-effectiveness boundary, further indicating that the economic difference between the two drugs was modest. The probabilistic sensitivity analysis results are shown in Figures 2, 3, and the probabilities of each strategy being cost-effective at the prespecified threshold are presented in Supplementary Figure 1.

**Figure 2:**
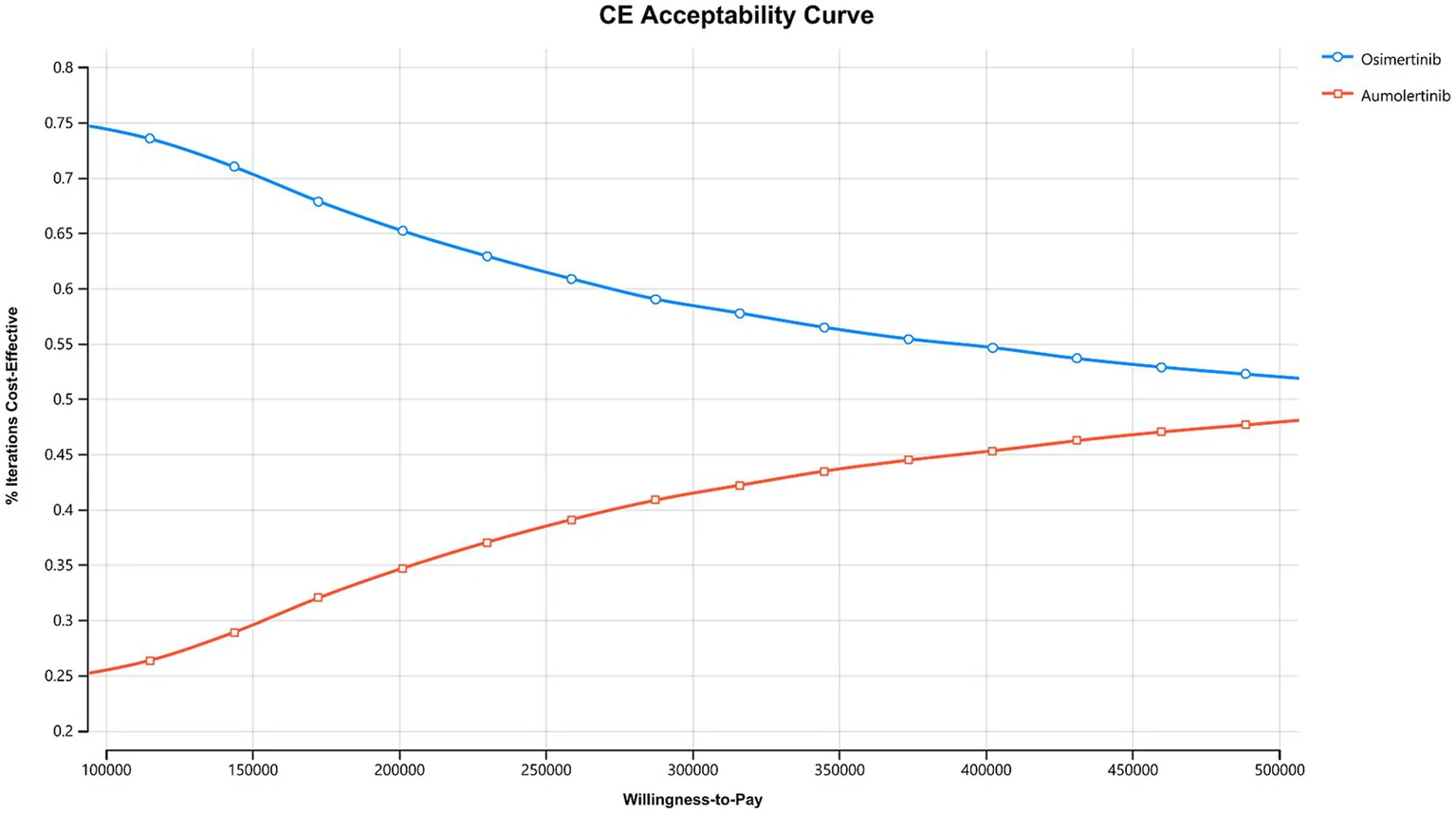
Cost-effectiveness acceptability curve for aumolertinib versus osimertinib. The curve shows the probability that each strategy is cost-effective across a range of willingness-to-pay thresholds. The prespecified base-case threshold in this study was 298,995 CNY/QALY. CE, Cost-effectiveness.

**Figure 3:**
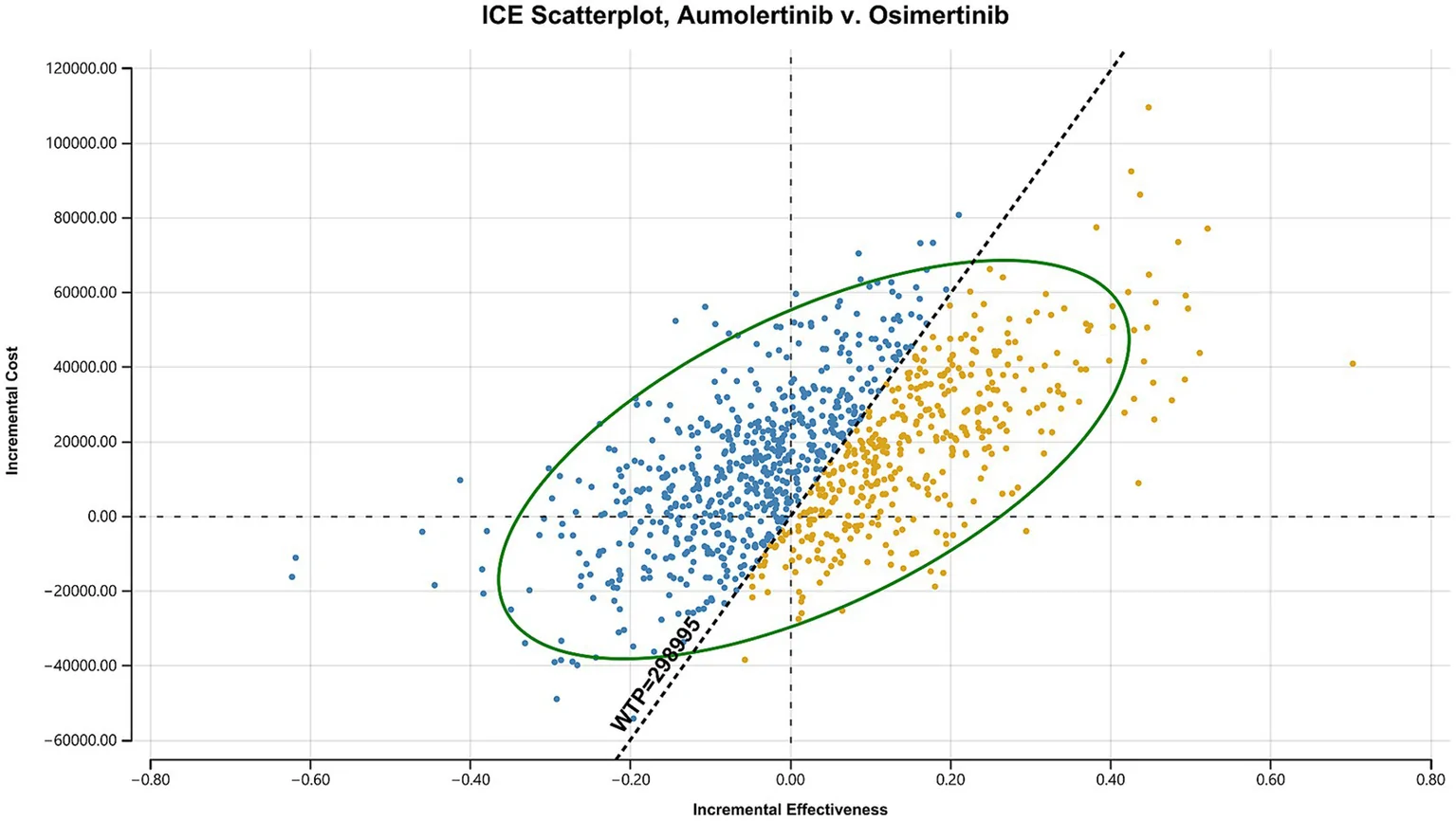
Incremental cost-effectiveness scatter plot for aumolertinib versus osimertinib. Each point represents one probabilistic simulation. The dashed diagonal line indicates the willingness-to-pay threshold of 298,995 CNY/QALY. Points below the line indicate aumolertinib is cost-effective.

### 3.3 Scenario and threshold analyses

Scenario analyses showed that the economic conclusion was sensitive to changes in drug price. When the price of osimertinib was reduced by 20%, the iNMB of aumolertinib versus osimertinib decreased to −30,039.72 CNY, and the probability of aumolertinib being cost-effective decreased to 21.5%. In contrast, when the price of aumolertinib was reduced by 20%, the iNMB increased to 16,448.50 CNY, and the probability of aumolertinib being cost-effective increased to 65.7%. Detailed scenario analysis results are shown in Table 4. In the OS HR threshold analysis, iNMB decreased monotonically as the OS HR increased and was approximately zero at an OS HR of 0.934. Thus, at the prespecified WTP threshold, aumolertinib was economically favorable at OS HRs below approximately 0.934, whereas osimertinib was favored at higher values within the evaluated range (Supplementary Figure 11).

**Table 4:** Scenario analyses.

Scenario	iNMB (CNY)	CE probability, aumolertinib
Base case	−8307.60	0.419
Osimertinib price reduced by 20%	−30039.72	0.215
Aumolertinib price reduced by 20%	16448.50	0.657

iNMB, incremental net monetary benefit; CNY, Chinese Yuan; CE, Cost-effectiveness.

### 3.4 Exploratory subgroup analyses

In the exploratory subgroup analyses, aumolertinib generated higher total QALYs than osimertinib in the Asian subgroup (1.918 vs. 1.804), the Ex19del subgroup (2.291 vs. 2.204), and the CNS metastases subgroup (1.996 vs. 1.837), but lower total QALYs in the L858R subgroup (1.567 vs. 1.666). The incremental cost of aumolertinib ranged from 1,475.17 CNY in the L858R subgroup to 24,355.77 CNY in the CNS metastases subgroup. At the prespecified threshold, the corresponding iNMB values were 13,354.74 CNY for the Asian subgroup, 4,192.45 CNY for the Ex19del subgroup, −31,150.57 CNY for the L858R subgroup, and 23,319.18 CNY for the CNS metastases subgroup. Detailed subgroup results are presented in Table 5.

**Table 5:** Subgroup cost-utility results for aumolertinib versus osimertinib.

Analysis set	Treatment	Cost (CNY)	incrCost (CNY)	QALYs	incrQALYs	NMB (CNY)	iNMB (CNY)
Asian	Osimertinib	178,865.32	—	1.804	—	360,385.82	—
	Aumolertinib	199,764.64	20,899.32	1.918	0.115	373,740.56	13,354.74
Ex19del	Osimertinib	212,082.05	—	2.204	—	446,865.40	—
	Aumolertinib	233,885.93	21,803.89	2.291	0.087	451,057.85	4,192.45
L858R	Osimertinib	166,469.83	—	1.666	—	331,692.06	—
	Aumolertinib	167,945.00	1,475.17	1.567	−0.099	300,541.49	−31,150.57
CNS metastases	Osimertinib	182,790.45	—	1.837	—	366,416.60	—
	Aumolertinib	207,146.22	24,355.77	1.996	0.159	389,735.77	23,319.18

CNY, Chinese Yuan; QALY, quality-adjusted life-year; NMB, net monetary benefit; iNMB, incremental net monetary benefit; CNS, central nervous system.

The forest plot showed that, on point estimate, the CNS metastases subgroup was the most favorable to aumolertinib, whereas the L858R subgroup was the least favorable. However, the 95% uncertainty intervals for subgroup-specific iNMB crossed zero in all subgroups, including the Asian subgroup (−52,138 to 100,310 CNY), the Ex19del subgroup (−83,222 to 100,768 CNY), the L858R subgroup (−103,633 to 62,785 CNY), and the CNS metastases subgroup (−69,932 to 159,627 CNY), indicating substantial uncertainty in all exploratory subgroup analyses. The forest plot is shown in Figure 4.

**Figure 4:**
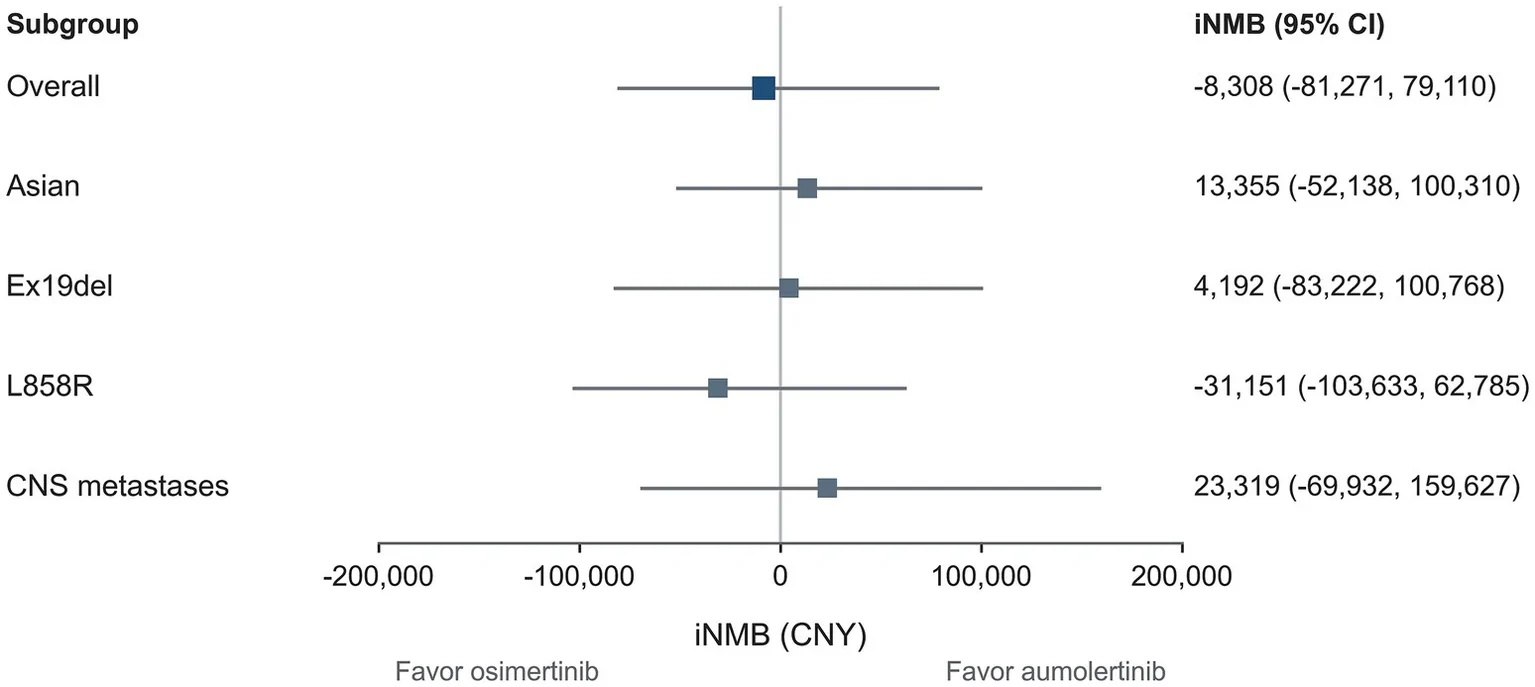
Forest plot of subgroup-specific incremental net monetary benefit. Points indicate subgroup-specific iNMB estimates and horizontal lines indicate 95% uncertainty intervals. Values to the right of zero favor aumolertinib, whereas values to the left favor osimertinib.

The directional pattern observed in subgroup probabilistic sensitivity analyses was generally consistent with the subgroup iNMB point estimates. At the prespecified threshold, the probability of aumolertinib being cost-effective was 62.23% in the Asian subgroup, 51.82% in the Ex19del subgroup, 23.62% in the L858R subgroup, and 64.52% in the CNS metastases subgroup. Overall, the subgroup point estimates differed in direction across molecular and clinical subgroups, although all subgroup results should be interpreted as exploratory because the OS HR was conservatively fixed at 1.00 in all subgroup analyses. Detailed subgroup probabilistic sensitivity analysis results are shown in Supplementary Figures 7–10.

## 4 Discussion

In this cost-utility analysis conducted from the perspective of the Chinese healthcare system, aumolertinib generated slightly higher QALYs than osimertinib in the overall population, but it was also associated with higher total costs. At the prespecified WTP threshold of 298,995 CNY/QALY, the iNMB of aumolertinib versus osimertinib was negative, indicating that aumolertinib was not economically favorable in the base-case analysis. However, the absolute difference between the two strategies was small, with an incremental gain of only 0.019 QALYs and an incremental cost of 14,037.26 CNY. Taken together, these findings suggest that the two third-generation EGFR-TKIs are economically similar overall, rather than clearly separated in favor of either strategy.

In the absence of a head-to-head randomized trial, comparative efficacy remains an important source of uncertainty. For PFS, however, the available indirect evidence was notably consistent: the STC, conventional Bucher comparison, and two subsequent Bayesian NMAs yielded aumolertinib-versus-osimertinib HRs of 0.98, 1.00, 1.03, and 1.02, respectively (20, 22, 23) (Supplementary Table 3). Although these estimates are not independent clinical replications, their close agreement across analytical approaches supports the use of a near-neutral comparative PFS effect.

Greater uncertainty remains for OS. The early STC estimate was based on immature AENEAS OS data, whereas updated long-term AENEAS data and supportive Bucher comparisons using FLAURA overall and FLAURA China produced point estimates closer to unity (10, 24, 25) (Supplementary Table 3). These findings support the use of OS HR = 1.00 as a neutral modeling assumption, while the wide uncertainty around the indirect estimates precludes a definitive comparative OS conclusion. The threshold analysis showed that the economic preference changed at an OS HR of approximately 0.934, highlighting the importance of this remaining uncertainty.

Earlier studies suggested that, under previous pricing conditions, first-line osimertinib was not cost-effective compared with first-generation EGFR-TKIs in China (13, 14). In the analysis by Li et al. (13), osimertinib was estimated to require a price reduction of 50% to reach the cost-effectiveness threshold, whereas Shu et al. (14), using a later and already substantially reduced market price, still concluded that an additional price reduction of more than 5% would be needed for osimertinib to become cost-effective in China. In contrast, Zhang et al. (16) reported that aumolertinib was cost-effective compared with gefitinib, and drug cost was the most influential parameter in that model. These studies differed in comparators, model structures, time horizons, price years, and outcome measures, so direct quantitative comparison is not appropriate. Nevertheless, they could reasonably create an expectation that aumolertinib might be more likely to show an economic advantage over osimertinib in China. Our findings did not confirm that expectation.

One plausible explanation is that the price of osimertinib has declined substantially over time (31, 32). In the present model, the per-cycle cost of osimertinib was 4,635.12 CNY per 28 days. This cost was lower than those reported in earlier Chinese pharmacoeconomic studies of osimertinib (13, 14), suggesting that the current pricing environment may have substantially narrowed the economic gap between the two strategies. The scenario analyses further support this interpretation, as a moderate price reduction for either drug was sufficient to materially change the probability of being cost-effective.

This interpretation also has an important policy implication. When differences in clinical benefit are small, the relative cost-effectiveness of competing targeted therapies may become highly sensitive to drug price, such that even modest price adjustments can reverse the economic conclusion. From this perspective, our findings do not suggest that aumolertinib lacks economic competitiveness in a broader sense. Rather, they indicate that, within the current Chinese pricing framework, the existing price gap between aumolertinib and osimertinib is not large enough to give aumolertinib a clear economic advantage. Because periodic National Reimbursement Drug List negotiations and other reimbursement-related price adjustments may change the acquisition prices of the two drugs by different magnitudes or at different times, their relative price gap and consequently the economically preferred strategy may change over time. The present findings should therefore be interpreted as conditional on current prices, and re-evaluation may be warranted after material changes in either drug price.

Another notable finding of this study was the variation in modeled economic outcomes across exploratory subgroups. Aumolertinib showed a numerically positive iNMB in the Asian, Ex19del, and CNS metastases subgroups, whereas its iNMB was negative in the L858R subgroup. These results suggest that the balance between incremental benefit and incremental cost may differ across clinical populations. Such directional differences across mutation-defined subgroups may not be entirely unexpected, as previous studies have suggested that clinical outcomes after EGFR-TKI treatment can differ between patients with exon 19 deletion and those with L858R mutation (33, 34). However, these subgroup findings should be interpreted with considerable caution. The 95% uncertainty intervals crossed zero in all subgroups, and all subgroup analyses were conducted under the same conservative assumption of no OS difference. In addition, although the CNS metastases subgroup used a subgroup-specific PFS anchor curve, its OS component was still anchored to the overall-population curve because CNS-specific OS data were unavailable. These subgroup analyses are therefore better viewed as exploratory assessments of subgroup-specific economic patterns rather than evidence sufficient to support subgroup-specific decision-making.

This study has several strengths. First, to our knowledge, it is the first pharmacoeconomic evaluation conducted within the Chinese healthcare setting to specifically compare these two third-generation EGFR-TKIs. Second, the model used a partitioned survival framework that is widely applied in oncology economic evaluations and incorporated direct medical costs relevant to the Chinese healthcare system. Third, the analysis not only compared the overall economic outcomes of the two strategies, but also highlighted a clinically and policy-relevant issue: when survival differences are small, drug cost can become a major determinant of comparative cost-effectiveness. Finally, where feasible, subgroup-specific anchor curves were incorporated into the exploratory subgroup analyses, allowing subgroup-specific economic patterns to be explored for future evaluation.

Several limitations should also be acknowledged. First, this study was based on indirect comparison rather than head-to-head trial evidence, and its validity therefore depends on the assumptions underlying the STC and Bucher methods, including the assumption that the comparator arms in the source studies were sufficiently comparable. Second, the comparative OS effect between aumolertinib and osimertinib remained uncertain because available estimates were based on indirect cross-trial comparisons. The OS HR was therefore set at 1.00 in the overall and subgroup analyses, which may have attenuated a true long-term difference between the two drugs. Third, survival inputs were reconstructed from published Kaplan–Meier curves rather than derived from original individual patient data, which inevitably introduced additional uncertainty into model calibration and extrapolation. Fourth, the CNS metastases subgroup analysis was only partially subgroup-specific because its PFS anchor was subgroup-specific whereas its OS anchor remained based on the overall population. Finally, health state utilities were based on China-specific estimates from an external time trade-off study rather than being measured directly in the relevant trial populations. Although this choice improved regional relevance, these values may still not fully capture trial-specific health-related quality of life. AE-specific disutilities were also not separately modeled. This simplification may result in modest differences in estimated QALYs and may not fully capture subtle differences in tolerability between the two treatments.

Future research should focus on reducing these major sources of uncertainty. Direct comparative evidence between aumolertinib and osimertinib would provide the strongest basis for economic evaluation. In the absence of such evidence, additional long-term comparative OS evidence, more robust subgroup survival data, and real-world data from Chinese clinical practice would all improve future models. In particular, real-world evidence from Chinese cohorts could help address the current evidence gap in patients with CNS metastases by providing subgroup-specific information on long-term survival, intracranial progression, and post-progression treatment sequences, thereby reducing reliance on the overall-population OS anchor. Once more robust subgroup-specific OS information becomes available, the exploratory findings reported here should be revisited to determine whether the subgroup patterns observed in this study persist within a less restrictive evidence framework. Overall, the present study suggests that, under the current Chinese pricing environment and available comparative evidence, aumolertinib and osimertinib have broadly similar economic profiles in the overall population, with the base-case analysis slightly favoring osimertinib at the prespecified threshold. The exploratory subgroup analyses showed variation in point estimates across subgroups, but the uncertainty remains substantial and these findings should not be overinterpreted.

## 5 Conclusion

From the perspective of the Chinese healthcare system, aumolertinib and osimertinib showed broadly similar economic profiles as first-line treatment for advanced EGFR-mutated NSCLC. In the base-case analysis, aumolertinib was associated with a small incremental health gain but higher cost, and therefore was not economically favorable relative to osimertinib at the prespecified WTP threshold. Exploratory subgroup analyses showed variation in point estimates across clinical populations, but these findings remained highly uncertain and should be interpreted with caution. Overall, the present study suggests that, under the current pricing environment and available comparative evidence, neither strategy showed a clear economic advantage in the overall population.

## Data Availability

The original contributions presented in the study are included in the article/Supplementary material, further inquiries can be directed to the corresponding authors.
