# Supplementary material for: Cost-effectiveness of first-line aumolertinib versus osimertinib for advanced epidermal growth factor receptor-mutated non-small cell lung cancer in China
Source: Front Public Health. 2026 Sep 22;14:1858476. doi: 10.3389/fpubh.2026.1858476 (PMC13638405; doi:10.3389/fpubh.2026.1858476)
Supplement: Supplementary file 1 [file Data_Sheet_1.DOCX]

Supplementary Material

**Supplementary Table 1. Parameter inputs for subsequent treatment after disease progression**

| **Parameter** | **Value** | **Source** |
| --- | --- | --- |
| **Subsequent treatment uptake after progression** |  |  |
| Proportion of progressed patients receiving subsequent treatment | 0.61 | (1) |
| **Treatment mix among patients receiving subsequent treatment** |  |  |
| Proportion receiving platinum-based chemotherapy | 0.70 | (1) |
| Proportion receiving other EGFR-TKIs | 0.30 | (1) |
| **Drug prices for representative subsequent regimens (CNY)** |  |  |
| Pemetrexed/0.1g | 98.00 | Drug procurement platform |
| Pemetrexed/0.5g | 335.98 | Drug procurement platform |
| Cisplatin/30mg | 19.12 | Drug procurement platform |
| Gefitinib/0.25g | 11.85 | Drug procurement platform |
| **Treatment duration assumptions (months)** |  |  |
| Median duration of platinum-based chemotherapy | 3.10 | (2) |
| Median duration of other EGFR-TKIs treatment | 5.70 | (3) |
| **Patient characteristic used for chemotherapy dosing** |  |  |
| Height (m) | 1.64 | Assumption |
| Weight (kg) | 65.00 | Assumption |

The weighted average cost of subsequent treatment was estimated as the proportion of patients receiving active post-progression treatment multiplied by the expected cost of representative subsequent regimens weighted according to the simplified treatment mix. Platinum-based chemotherapy was represented by pemetrexed plus cisplatin, and other EGFR-TKIs were represented by gefitinib. The total cost of each representative regimen was estimated on the basis of the reported dosing schedule and median treatment duration. The resulting weighted average cost was applied in the model as a one-time cost at the time of progression. EGFR-TKI, epidermal growth factor receptor–tyrosine kinase inhibitor; CNY, Chinese Yuan.

**Supplementary Table 2.** **Key design and population characteristics of the AENEAS and FLAURA trials (1, 4, 5)**

| **Characteristic** | **AENEAS** | **FLAURA** |
| --- | --- | --- |
| Trial identifier | NCT03849768 | NCT02296125 |
| Trial design | Multicenter, randomized, double-blind, phase III trial | Multicenter, randomized, double-blind, phase III trial |
| Geographic setting | 53 sites in mainland China | 132 sites in 29 countries |
| Enrollment period | November 2018 to September 2019 | December 2014 to March 2016 |
| Randomized population | 429 patients: aumolertinib, n=214; gefitinib, n=215 | 556 patients: osimertinib, n=279; standard EGFR-TKI, n=277 |
| Disease setting | Previously untreated locally advanced or metastatic EGFR-mutated NSCLC | Previously untreated locally advanced or metastatic EGFR-mutated NSCLC |
| Eligible EGFR mutations | Ex19del or L858R | Ex19del or L858R |
| EGFR mutation ascertainment | Central laboratory testing; tissue preferred, with blood permitted | Local or central testing |
| Experimental treatment | Aumolertinib 110 mg orally once daily | Osimertinib 80 mg orally once daily |
| Comparator treatment | Gefitinib 250 mg orally once daily | Gefitinib 250 mg once daily or erlotinib 150 mg once daily |
| Comparator composition | Gefitinib only | Gefitinib, 66%; erlotinib, 34% |
| Randomization ratio | 1:1 | 1:1 |
| Randomization stratification factors | EGFR mutation type and CNS metastases status | EGFR mutation type and race |
| Primary endpoint | Investigator-assessed PFS according to RECIST 1.1 | Investigator-assessed PFS according to RECIST 1.1 |
| Median age, years | 59 vs. 62 | 64 vs. 64 |
| Female, % | 62.6 vs. 62.8 | 64 vs. 62 |
| Asian race, % | 100 vs. 100; study population was ethnic Chinese | 62 vs. 62 |
| Never-smoker, % | 72.9 vs. 67.0 | 65 vs. 63 |
| Performance status | ECOG PS 0: 23.8% vs. 25.1%; ECOG PS 1: 74.8% vs. 74.0% | WHO PS 0: 40% vs. 42%; WHO PS 1: 60% vs. 58% |
| Ex19del, % | 65.4 vs. 65.6 | 63 vs. 63 |
| L858R, % | 34.6 vs. 34.4 | 37 vs. 37 |
| CNS metastases at baseline, % | 26.2 vs. 27.4 | 19 vs. 23 |
| Advanced disease status | Stage IV: 94.4% vs. 92.1% | Metastatic disease: 95% vs. 95% |
| Eligibility of patients with CNS metastases | Patients with asymptomatic, stable CNS metastases were eligible; no steroid requirement for ≥2 weeks before study treatment | Patients with neurologically stable CNS metastases were eligible; previous definitive treatment or glucocorticoid therapy was required to have been completed ≥2 weeks before study treatment |
| Tumor-assessment schedule | Baseline and every 6 weeks (±7 days) through month 15, then every 12 weeks | Baseline and every 6 weeks (±1 week) through month 18, then every 12 weeks until progression |
| Separate maintenance phase | None; randomized EGFR-TKI treatment was administered continuously | None; randomized EGFR-TKI treatment was administered continuously |
| Treatment duration | Until disease progression, unacceptable toxicity, withdrawal of consent, or other protocol-defined discontinuation criteria | Until disease progression, unacceptable toxicity, or withdrawal of consent |
| Treatment beyond RECIST progression | Permitted if continued clinical benefit was judged by the investigator | Permitted if continued clinical benefit was judged by the investigator |
| Crossover rule | Patients receiving gefitinib who developed disease progression and acquired T790M were eligible to cross over to aumolertinib | Patients receiving standard EGFR-TKI could cross over to open-label osimertinib after confirmed disease progression and post-progression confirmation of T790M positivity; intervening anticancer therapy was not permitted before crossover |
| Observed crossover | 41/215 (19.1%) patients in the gefitinib arm had received crossover aumolertinib by the January 15, 2021 cutoff | At the primary PFS cutoff, 48 patients crossed over within the trial; at the final OS analysis, 70 patients had crossed over within the trial and an additional 15 had received osimertinib outside the trial |
| Subsequent treatment after progression | Detailed distribution of non-crossover subsequent therapies was not reported in the available primary publication | Subsequent therapies were reported in detail; at the final OS analysis, 133/279 (48%) patients in the osimertinib arm and 180/277 (65%) in the comparator arm received a first subsequent anticancer therapy |
| Median follow-up for primary PFS analysis | 20.5 months with aumolertinib and 20.7 months with gefitinib | Median PFS follow-up 15.0 months with osimertinib and 9.7 months with standard EGFR-TKI |
| Final OS follow-up | Mature final OS analysis was not available in the AENEAS publication used for the indirect comparison | Median OS follow-up 35.8 months with osimertinib and 27.0 months with comparator EGFR-TKI |

**Supplementary Table 3. Comparative efficacy evidence for aumolertinib versus osimertinib**

| **Endpoint** | **HR (95% CI/CrI)** | **Evidence description** | **Source** |
| --- | --- | --- | --- |
| PFS | 0.98 (0.68–1.42) | Anchored STC using AENEAS patient-level data and published FLAURA aggregate data | (6) |
| PFS | 1.00 (0.72–1.39) | Conventional anchored Bucher comparison based on AENEAS and FLAURA | (6) |
| PFS | 1.03 (0.90–1.18) | Bayesian NMA of 7 phase III RCTs (n=3,012) | (7) |
| PFS | 1.02 (0.74–1.39) | Bayesian NMA of 11 phase III RCTs (n=4,663) | (8) |
| OS | 0.73 (0.44–1.22) | STC based on AENEAS OS data that were immature at the time of analysis | (6) |
| OS | 1.02 (0.67–1.56) | Conventional anchored Bucher comparison based on AENEAS and FLAURA | (6) |
| OS | 0.848 (0.677–1.062) | Updated long-term AENEAS OS; aumolertinib versus gefitinib | (9) |
| OS | 1.06 (0.77–1.45) | Bucher comparison using updated AENEAS OS and FLAURA overall OS | (1, 9), author calculation |
| OS | 1.00 (0.62–1.60) | Bucher comparison using updated AENEAS OS and FLAURA China OS | (9, 10), author calculation |

HRs are expressed as aumolertinib versus osimertinib except for the updated AENEAS source-trial estimate, which compares aumolertinib with gefitinib. For the two updated Bucher comparisons, HRs and approximate 95% CIs were calculated by the authors on the log-HR scale using published trial-level estimates. CI, confidence interval; CrI, credible interval; PFS, progression-free survival; OS, overall survival; STC, simulated treatment comparison; NMA, network meta-analysis.

**Supplementary Table 4. Selected parametric survival distributions for each anchor curve and analysis set**

| **Analysis set** | **Curve** | **Selected distribution** | **Parameter 1** | **Parameter 2** | **AIC** | **BIC** |
| --- | --- | --- | --- | --- | --- | --- |
| **Overall** | PFS | Log-Normal | 2.90 | 0.88 | 1116.00 | 1123.00 |
| **Overall** | OS | Gamma | 2.04 | 0.05 | 1526.00 | 1533.00 |
| **Asian** | PFS | Log-Normal | 2.82 | 0.77 | 666.00 | 673.00 |
| **Asian** | OS | Gamma | 2.67 | 0.06 | 972.00 | 979.00 |
| **Ex19del** | PFS | Log-Normal | 3.03 | 0.92 | 647.00 | 656.00 |
| **Ex19del** | OS | Gamma | 2.32 | 0.05 | 871.00 | 877.00 |
| **L858R** | PFS | Log-Normal | 2.70 | 0.86 | 454.00 | 459.00 |
| **L858R** | OS | Gamma | 1.83 | 0.05 | 648.00 | 654.00 |
| **CNS metastases** | PFS | Log-Normal | 2.72 | 0.86 | 227.00 | 231.00 |

For the parametric distributions reported in this table, Parameter 1 and Parameter 2 correspond to distribution-specific parameters as follows: for the log-normal distribution, μ and σ; and for the gamma distribution, α and λ. Parametric distributions were selected on the basis of statistical fit and visual inspection. For the CNS metastases subgroup, no subgroup-specific OS Kaplan–Meier curve or mature OS data were available; therefore, no separate CNS-specific OS distribution was estimated for this analysis set. In the model, the OS component for the CNS metastases subgroup was anchored to the overall-population OS curve, whereas the PFS component remained anchored to the CNS-specific curve. AIC, Akaike information criterion; BIC, Bayesian information criterion; PFS, progression-free survival; OS, overall survival; CNS, central nervous system.

**Supplementary Table 5. Derivation of first-line drug acquisition costs per 28-day model cycle**

| **Drug** | **Strength and package size** | **Procurement price per package (CNY)** | **Cost per tablet (CNY)** | **Daily dose** | **Requirement per cycle** | **Costs per cycle (CNY)** |
| --- | --- | --- | --- | --- | --- | --- |
| Osimertinib | 80 mg × 30 tablets/package | 4,966.20 | 165.54 | 80 mg once daily | 28 tablets | 4,635.12 |
| Aumolertinib | 55 mg × 20 tablets/package | 1,854.80 | 92.74 | 110 mg once daily | 56 tablets | 5,193.44 |

Per-cycle acquisition cost was calculated as procurement price per package ÷ number of tablets per package × number of tablets required per 28-day cycle. Costs were calculated on a per-tablet basis; fractional package-equivalents across cycles were permitted and package wastage was not modeled. CNY, Chinese Yuan.

**Supplementary Figure 1. Probability of each strategy being cost-effective at the prespecified WTP threshold in the overall population.** At the WTP threshold of 298,995 CNY/QALY, osimertinib had a cost-effectiveness probability of 0.58 and aumolertinib had a probability of 0.42.

**Supplementary Figure 2. Parametric survival fitting for the overall anchor curves.** Kaplan–Meier curves from the overall anchor population are shown together with fitted parametric survival models. Final distributions selected for extrapolation are reported in Supplementary Table 4.

**Supplementary Figure 3. Parametric survival fitting for the Asian subgroup anchor curves.** Kaplan–Meier curves from the FLAURA Asian subgroup are shown together with fitted parametric survival models. Final distributions selected for extrapolation are reported in Supplementary Table 4.

**Supplementary Figure 4. Parametric survival fitting for the Ex19del subgroup anchor curves.** Kaplan–Meier curves from the FLAURA Ex19del subgroup are shown together with fitted parametric survival models. Final distributions selected for extrapolation are reported in Supplementary Table 4.

**Supplementary Figure 5. Parametric survival fitting for the L858R subgroup anchor curves.** Kaplan–Meier curves from the FLAURA L858R subgroup are shown together with fitted parametric survival models. Final distributions selected for extrapolation are reported in Supplementary Table 4.

**Supplementary Figure 6. Parametric survival fitting for the CNS metastases subgroup anchor curves.** Published Kaplan–Meier curves from the FLAURA CNS metastases subgroup are shown together with candidate parametric survival functions and the final selected fitted curves for PFS. No separate CNS-specific OS curve was fitted because subgroup-specific OS Kaplan–Meier data were unavailable. In the model, the OS component for this subgroup was anchored to the overall-population OS curve. The selected distributions are reported in Supplementary Table 4.

**Supplementary Figure 7. Probabilistic sensitivity analysis for the Asian subgroup.** The upper-left panel shows the cost-effectiveness acceptability curve, the upper-right panel shows the probability of each strategy being cost-effective at the prespecified WTP threshold of 298,995 CNY/QALY, and the lower panel shows the incremental cost-effectiveness scatter plot.

**Supplementary Figure 8. Probabilistic sensitivity analysis for the Ex19del subgroup.** The upper-left panel shows the cost-effectiveness acceptability curve, the upper-right panel shows the probability of each strategy being cost-effective at the prespecified WTP threshold of 298,995 CNY/QALY, and the lower panel shows the incremental cost-effectiveness scatter plot.

**Supplementary Figure 9. Probabilistic sensitivity analysis for the L858R subgroup.** The upper-left panel shows the cost-effectiveness acceptability curve, the upper-right panel shows the probability of each strategy being cost-effective at the prespecified WTP threshold of 298,995 CNY/QALY, and the lower panel shows the incremental cost-effectiveness scatter plot.

**Supplementary Figure 10. Probabilistic sensitivity analysis for the CNS metastases subgroup.** The upper-left panel shows the cost-effectiveness acceptability curve, the upper-right panel shows the probability of each strategy being cost-effective at the prespecified WTP threshold of 298,995 CNY/QALY, and the lower panel shows the incremental cost-effectiveness scatter plot.

**Supplementary Figure 11. Threshold analysis of the overall survival hazard ratio.** The curve shows the iNMB of aumolertinib versus osimertinib across OS HRs from 0.40 to 1.30. The WTP threshold was 298,995 CNY/QALY, and all other model inputs were held constant. Positive iNMB values favor aumolertinib, whereas negative values favor osimertinib; the horizontal line at 0 CNY indicates equal NMB. The dashed vertical line marks the approximate threshold at an OS HR of 0.934, where iNMB was approximately zero. The base-case assumption (OS HR = 1.00; iNMB = −8,307.60 CNY) is also indicated.

**References**

1.	Ramalingam SS, Vansteenkiste J, Planchard D, Cho BC, Gray JE, Ohe Y, et al. Overall Survival with Osimertinib in Untreated, Egfr-Mutated Advanced Nsclc. *N Engl J Med* (2020) 382(1):41-50. doi: 10.1056/NEJMoa1913662.

2.	Mok TS, Wu Y-L, Ahn M-J, Garassino MC, Kim HR, Ramalingam SS, et al. Osimertinib or Platinum – Pemetrexed in Egfr T790m – Positive Lung Cancer. *N Engl J Med* (2017) 376(7):629-40. doi: 10.1056/NEJMoa1612674.

3.	Khoo T, Gao L. Cost-Effectiveness of Osimertinib Versus Standard Egfr-Tki as First-Line Treatment for Locally Advanced or Metastatic Egfr Mutation-Positive Non-Small Cell Lung Cancer in Australia. *Expert Rev Pharmacoecon Outcomes Res* (2021) 21(3):415-23. Epub 20201206. doi: 10.1080/14737167.2021.1847648.

4.	Soria J-C, Ohe Y, Vansteenkiste J, Reungwetwattana T, Chewaskulyong B, Lee KH, et al. Osimertinib in Untreated Egfr-Mutated Advanced Non–Small-Cell Lung Cancer. *N Engl J Med* (2018) 378(2):113-25. doi: 10.1056/NEJMoa1713137.

5.	Lu S, Dong X, Jian H, Chen J, Chen G, Sun Y, et al. Aeneas: A Randomized Phase Iii Trial of Aumolertinib Versus Gefitinib as First-Line Therapy for Locally Advanced or Metastatic Non–Small-Cell Lung Cancer with Egfr Exon 19 Deletion or L858r Mutations. *J Clin Oncol* (2022) 40(27):3162-71. doi: 10.1200/jco.21.02641.

6.	Popat S, Stergiopoulos S, King S, Curtis M, Dillon A, Akehurst R, et al. 1005p Indirect Treatment Comparisons of Relative Efficacy for Aumolertinib Vs Osimertinib in Egfr-Positive Non-Small Cell Lung Cancer (Nsclc). *Ann Oncol* (2022) 33:S1012-S3. doi: 10.1016/j.annonc.2022.07.1131.

7.	Du W, Li A, Xiao B, Yang Y, Fang W, Huang Y, et al. First-Line Third-Generation Egfr Tyrosine Kinase Inhibitor Monotherapy for Advanced Egfr-Mutated Non-Small Cell Lung Cancer: A Systematic Review and Network Meta-Analysis. *MedComm (2020)* (2025) 6(11):e70393. Epub 20251106. doi: 10.1002/mco2.70393.

8.	Liu X, Zhao Q, Sun W, Chen J. First-Line Third-Generation Egfr-Tkis for Advanced Egfr-Mutated Non-Small Cell Lung Cancer: A Systematic Review and Network Meta-Analysis of Randomized Controlled Trials. *J Thorac Dis* (2026) 18(6):617. Epub 20260521. doi: 10.21037/jtd-2026-1-0317.

9.	Taiwan Food and Drug Administration. *Assessment Report: Pulmivex Film-Coated Tablets 55 Mg (Aumolertinib Mesilate)*. Taiwan: Taiwan Food and Drug Administration (2026).

10.	Cheng Y, He Y, Li W, Zhang HL, Zhou Q, Wang B, et al. Osimertinib Versus Comparator Egfr Tki as First-Line Treatment for Egfr-Mutated Advanced Nsclc: Flaura China, a Randomized Study. *Target Oncol* (2021) 16(2):165-76. Epub 20210205. doi: 10.1007/s11523-021-00794-6.
